# Analysis of serum homocysteine in the laboratory practice - comparison of the direct chemiluminescence immunoassay and high performance liquid chromatography coupled with fluorescent detection

**DOI:** 10.11613/BM.2020.030703

**Published:** 2020-08-05

**Authors:** Łukasz Paprotny, Dorota Wianowska, Magdalena Izdebska, Agnieszka Celejewska, Dorota Szewczak, Janusz Solski

**Affiliations:** 1 Research and Development Center, Alab Laboratories, Lublin, Poland; 2 Department of Chromatographic Methods, Faculty of Chemistry, Maria Curie-Skłodowska University, Lublin, Poland; 3 Department of Laboratory Diagnostics, Medical University of Lublin, Lublin, Poland

**Keywords:** homocysteine, immunoassay, luminescent measurements, liquid chromatography

## Abstract

**Introduction:**

Effective diagnosis of cardiovascular diseases requires the right tools to be used enabling selective and sensitive analysis of their biomarkers. One of them is homocysteine (Hcy), nowadays determined by immunoassays and chromatographic methods. This study aims to compare the results obtained by direct chemiluminescence immunoassay (CLIA) and high performance liquid chromatography with fluorescent detection (HPLC-FD) using commercial kits.

**Materials and methods:**

Homocysteine concentration was determined in serum samples obtained from 101 individuals, using Atellica IM HCY (Siemens Healthineers, Erlangen, Germany) and HCY in plasma/serum – HPLC-FD (Chromsystems Instruments & Chemicals GmbH, Gräfelfing, Germany) tests validated for routine analysis. The latter was applied as a reference method. The comparability and agreement between the tested methods were evaluated using the Passing-Bablok (PB) regression analysis and the Bland-Altman (BA) method of the differences analysis.

**Results:**

Studies showed that CLIA gives higher Hcy concentrations (15.7 ± 4.14 μmol/L). Passing-Bablok regression analysis of the results obtained with CLIA (y) compared with HPLC-FD (x) yielded an intercept of 0.22 (95%CI: - 2.16 to 2.46) and slope of 1.58 (95%CI: 1.33 to 1.87). Bland-Altman analysis demonstrated a systematic positive bias for CLIA of 5.85 ± 2.77 µmol/L.

**Conclusions:**

Methods disagreement precludes their interchangeability. Lower Hcy values by HPLC-FD result from its greater selectivity. High performance liquid chromatography with fluorescent detection should be considered as preferential method for analysing Hcy in blood serum as well as the recommended reference method for routine clinical analysis. This fact, however, imposes the need to establish new reference ranges.

## Introduction

Cardiovascular diseases (CVD) are one of the major health problems in the modern world. The WHO data reveals that unfortunately, CVD are the main cause of people mortality (*1*). Therefore in the laboratory practice effective diagnostic tools are needed that allow for selective and sensitive analysis of cardiovascular diseases markers.

Many clinical and epidemiological studies have confirmed the significant relationship between hyperhomocysteinemia, *i.e.* a medical condition characterized by a high homocysteine in the blood and the risk of myocardial infarction in general population (*2*-*5*). Therefore homocysteine (Hcy), the sulphur amino acid formed during metabolism of methionine may be considered as a biomarker and its high level as a prognostic factor for cardiovascular events and mortality (*6*-*8*). It is worth noting that CVD patients with high total homocysteine (tHcy) concentrations (above 13-15 μmol/L) belong already to a high-risk group (especially with coronary artery disease, diabetes and renal failure) (*5*, *6*). There can be many causes of hyperhomocysteinemia. Some of them are nutritional deficiencies of folate, vitamins B_6_ and B_12_, and genetic defects of enzymes (particularly methylenetetrahydrofolate reductase (MTHFR) and cystathionine-β-synthase (CBS)) involved in Hcy metabolism (*3*). Scientific and clinical research confirmed also higher Hcy concentrations in the serum of patients with Alzheimer’s disease, Parkinson’s disease, type 2 diabetes, metabolic syndrome, cancers and psychiatric disorders. High Hcy concentrations are also related to pregnancy complications and birth defects (*4*, *5*). In view of the above, the determination of tHcy in serum is an important diagnostic test requiring appropriately sensitive, but also specific analytical methods.

Homocysteine determination can be performed by many analytical techniques. The routinely used diagnostic methods are immunoassays, such as enzyme immunoassay (EIA), fluorescence polarization immunoassay (FPIA) and chemiluminescence immunoassay (CLIA) (*6*, *9*). However, recently the interest in the use of chromatographic methods in the diagnostic tests has increased (*10*). There are many chromatographic techniques. Yet of all, liquid chromatography methods coupled to various selective detectors such as a fluorescence detector (FD) or a mass spectrometric detector are most frequently applied. These methods combine the separation power of chromatographic columns and the specific identity of detection techniques so they are regarded as the gold analytical standard suitable for very sensitive and selective analysis of organic compounds in the complex biological matrices. Compared to the immunological methods, they are distinguished not only by high specificity but also the ability to analyse various compounds at very different concentration levels as well as in a wide concentration range, often during the same analysis (*9*, *11*, *12*). For these reasons, chromatographic methods are becoming increasingly popular and even recommended as reference methods in routine clinical analysis (*10*-*13*).

In the literature, one can find the data assessing the compatibility of the homocysteine determinations by immunological methods, mainly FPIA and EIA, with the chromatographic ones (*6*, *14*-*18*). According to the authors’ knowledge, however, there is no data on the assessment of the obtained results in terms of compliance of the direct chemiluminescence immunoassay method to the chromatographic method validated to the routine analysis. Therefore the aim of this study was to compare the compatibility of the homocysteine determinations by the CLIA method, routinely used in our laboratory, with the ready to use high performance liquid chromatography with fluorescent detection (HPLC-FD) method.

## Materials and methods

### Subjects

The serum samples used in the study (N = 101; 11 men and 90 women) were randomly selected from blood samples obtained from patients who underwent medical assessment of Hcy concentration during our laboratory standard practices (Alab Laboratories, Lublin, Poland). Blood was collected, in accordance with local and national regulations on ethics, from the patients only during their laboratory tests - no additional material was collected from the patients more than required for the medical evaluation of Hcy concentration.

The blood samples were obtained by fasting vein puncture, in the morning from 7.30 to 11 am. The blood was collected into the Sarstedt Monovette Homocysteine Z-Gel tubes containing a homocysteine stabilizer (SARSTEDT AG & Co. Nümbrecht, Germany), that provides stability for up to 96 hours if stored at room temperature, up to 1 month if refrigerated, and up to 10 months if frozen. The tubes have been tested to validate their suitability for the CLIA and HPLC-FD methods by the manufacturer (*19*). The serum was separated within 45 minutes of blood collection by centrifugation (2500 rpm; 5 min at room temperature) and frozen at - 20°C until testing. The samples were stored for up to three days prior to analysis.

### Methods

The tests were carried out using the standard diagnostic analysers Atellica IM 1600 (Siemens Healthineers, Erlangen, Germany) and the HPLC-FD system (NexeraX2, Shimadzu Corporation, Kyoto, Japan) under conditions indicated by the kit manufactures. The assays were carried out simultaneously by both methods to avoid any possible changes in Hcy and according to the instructions supplied by the manufactures of the respective kits.

The Atellica IM HCY test is a competitive immunoassay using direct chemiluminescence based on a three-step procedure. Firstly, a reducing reagent (dithiothreitol) is used to release Hcy bound to the plasma proteins. Then the total homocysteine, *i.e.* the sum of free Hcy as well as that released, is converted to S-adenosylhomocysteine (SAH) using an enzyme reagent (S-adenosylhomocysteine hydrolase). In a patient sample SAH competes with SAH covalently bound to the paramagnetic particles in a solid phase reagent, for a limited number of binding sites with the mouse monoclonal antibodies (anti-SAH) labelled with the acridine ester used as a chemiluminescent tracer. The amount of Hcy was calculated by relating the SAH chemiluminescence response to the SAH calibration curve obtained using the highly purified SAH calibrators (LOT no. 36852) supplied by the kit manufacture. Control materials from the kit manufacturer were used to carry out quality control (lot 36853).

The used HPLC-FD method is a multi-step procedure intended for the routine Hcy determination using a selective separation column as well as a selective fluorescence detector operating at two wavelengths (385 nm for excitation and 515 nm for emission). As in the previous method, the first stage involves reducing the plasma protein bound Hcy (using a reduction reagent - lot 4618) to a free form so that the total homocysteine can be determined. The next step, that distinguish this method from the previous one, is the precipitation of serum proteins using a precipitation reagent (lot 4618). The purified sample is subjected derivatization using a derivatizing reagent (lot 4618), followed by the HPLC-FD analysis. During the chromatographic analysis, the individual sample components are separated based on the differences in the affinity for the stationary phase (HPLC – column, order no. 39100) and the mobile phase (lot 2719) (both supplied by the kit manufacturer), which causes them to migrate at different rates to a detector where they are selectively and sensitively detected. The amount of Hcy was calculated by relating the Hcy chromatographic response to the Hcy calibration curve obtained using the highly purified Hcy calibrators (lot 0818) and the internal standard (lot 04618), both supplied by the kit manufacture. To check the correctness of the calibration, control tests at two homocysteine levels (Plasma Control Level I and Plasma Control Level II, lot 0919) were analysed. The correctness of the calibration was confirmed by obtaining results within the manufacturer’s declared range of homocysteine concentrations in the control samples.

### Statistical analysis

Method comparison was performed using the Passing-Bablok regression analysis and the Bland-Altman method for the differences analysis with an agreement indicator (bias between methods), recommended by the Clinical and Laboratory Standard Institute (CLSI) (*20*-*23*).

The Passing-Bablok regression analysis of the results is presented with a scatter diagram, a regression line, as well as a regression equation, where the intercept and slope represent respectively a constant difference interpreted as the systematic bias and the proportional measurement error between the methods. 95% confidence interval (CI) of the intercept and the slope explains if their value differ from value zero (intercept) and value one (slope). If 0 is in the CI of the intercept, and 1 is in the CI of the slope, the two methods are comparable within the investigated concentration range.

The Bland-Altman analysis of differences describes agreement between two quantitative methods by constructing limits of agreement. These limits are calculated by the mean and standard deviation (SD) of the differences between two measurements. If the differences are normally distributed the limits of agreement = the mean ± 1.96 SD (95% of differences is located between the mean - 1.96 SD and the mean + 1.96 SD). The mean difference is interpreted as the systematic bias.

The D’Agostino-Pearson test was used to check the normality of the differences between the tested methods. P < 0.05 was considered significant. The statistical analysis was carried out using the MedCalc Statistical Software Version 19.1.3 (Ostend, Belgium) and GraphPad Prism 8.30 (GraphPad Software, San Diego, USA).

## Results

**Figure 1:**
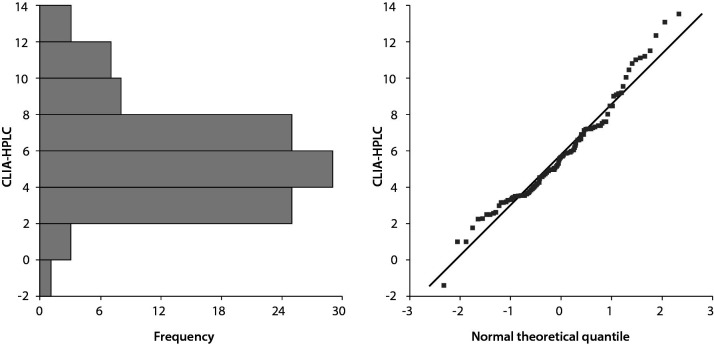
Distribution plots of differences between Hcy measurements by CLIA and HPLC-FD methods and Q-Q plots. CLIA - direct chemiluminiscence immunoassay. HPLC-FD - high-performance liquid chromatography with fluorescent detection.

**Figure 2:**
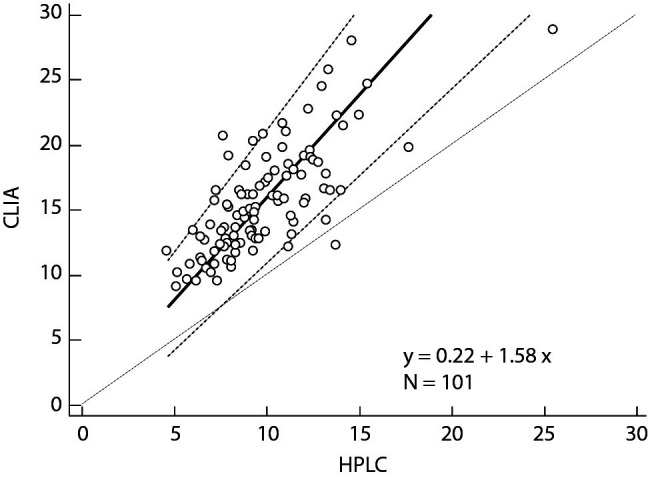
Passing-Bablok regression analysis of the results obtained with the direct chemiluminescence immunoassay (CLIA) compared with high-performance liquid chromatography with fluorescent detection (HPLC-FD). The dashed lines represent the 95% confidence intervals for the regression line. The identity line is represented as dotted.

Descriptive statistics of Hcy results obtained by the CLIA and HPLC-FD methods is presented in Table 1. Distribution plots of differences between Hcy measurements by CLIA and HPLC-FD methods and Q-Q plots are presented in Figure 1. The Passing-Bablok regression analysis of the results obtained with CLIA (y) compared with HPLC-FD (x) is presented in Figure 2. The comparison of the methods yielded the following equation: y = 0.22 + 1.58 x. 95% CI for the intercept is from - 2.16 to 2.46 and for the slop from 1.33 to 1.87. These results indicated proportional difference between methods.

**Table 1:** Descriptive statistics of homocysteine results obtained by the CLIA and HPLC-FD methods

	**Hcy concentrations (N = 101)**	
	**CLIA**	**HPLC-FD**
Mean ± SD	15.70 ± 4.14	9.86 ± 3.02
Minimum	9.10	4.63
Maximum	28.83	25.46
CLIA – direct chemiluminescence immunoassay. HPLC-FD – high performance liquid chromatography with fluorescent detection. Hcy – homocysteine. SD - standard deviation.		

**Figure 3:**
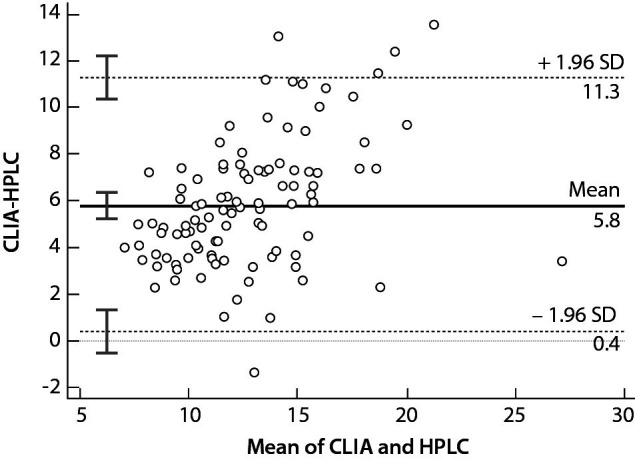
The Bland-Altman plots showing the difference in the Hcy results (µmol/L) between the two methods (CLIA and HPLC-FD), as a function of their mean value. Grey lines present confidence interval limits for mean and agreement limits. CLIA - chemiluminiscence immunassay. HPLC-FC - high-performance liquid chromatography with fluorescent detection.

**Figure 4:**
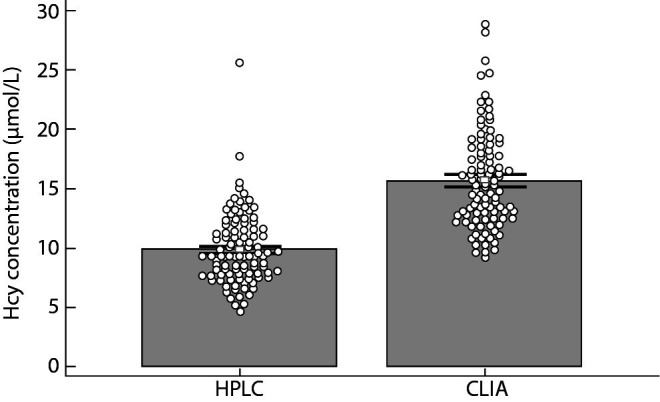
Graphical presentation of the Hcy measurements performed with the direct chemiluminescence immunoassay (CLIA) and high-performance liquid chromatography with fluorescent detection (HPLC-FD). Hcy – homocysteine.

The Bland-Altman difference plots are shown in Figure 3, and the results of statistical analysis in Table 2. It was found that the CLIA results for the whole tested range of Hcy concentrations were higher than the HPLC results. Graphical presentation of the tHcy measurements performed with the HPLC and CLIA methods is presented in Figure 4. The mean difference for CLIA vs HPLC-FD was 5.85 µmol/L (systematic positive bias) with a standard deviation (SD) of 2.77 (P < 0.001) (Figure 3).

**Table 2:** Bland-Altman statistical analysis of differences between serum tHcy concentrations measured by the CLIA compared with HPLC-FD methods

	**Hcy concentrations (N = 101)**
	**CLIA *vs* HPLC-FD**
Mean Difference	5.846
Standard Deviation	2.77
Standard Error	0.28
95% CI of the mean	5.298 to 6.394
95% Lower LoA	0.408
95% Upper LoA	11.284
CLIA – direct chemiluminescence immunoassay. HPLC-FD – high performance liquid chromatography with fluorescent detection. Hcy – homocysteine. CI – confidence interval. LoA – limit of agreement.	

## Discussion

This study compares the two analytical methods used in the laboratory practice, direct chemiluminescence immunoassay and high performance liquid chromatography with fluorescent detection, to determine the concentration of total homocysteine in the blood serum. HPLC-FD was chosen as the reference method. The obtained results showed that the tHcy concentrations measured by the CLIA method are higher than those obtained by HPLC-FD.

Chromatographic methods and especially those coupled with very specific and as a consequence very sensitive detectors, such as the fluorescence detector are widely recognized as very accurate and precise analytical tools. The HPLC-FD systems, due to the requirement of the derivatization of the analysed compound, in addition to the application of a separation column able to resolve a mixture of very similar compounds, guarantee a high level specificity of the determination. Moreover, the requirement for proper sample preparation prior to the analysis further increases the selectivity level. In turn, the application of the internal standard enhances the precision of the quantitative analysis. The immunoassays routinely used in laboratory diagnostics are sensitive and precise enough, but unfortunately their specificity is often lower compared to the chromatographic methods. The lower specificity of the CLIA method may therefore explain the observed higher homocysteine concentrations obtained by this method compared to the HPLC-FD method.

Comparative analysis performed to assess the agreement between the results obtained by the comparable methods indicated proportional difference and systematic positive bias. The systematic positive bias for CLIA suggests that the methodological differences of both methods may be responsible for the discrepancy between the tHcy results in the same patients group. Similar conclusions were drawn by the authors of other papers comparing immunoassays (*e.g.* ICL-Immulite 2000, Abbot IMx and Abbott AxSYM or Bio-Rad EIA immunoassays) with HPLC methods (*23*-*25*).

Demuth *et al.* for example, performed the evaluation of the agreement between the ADVIA Centaur and HPLC-FD methods (the latter was their in-house method developed based on the method previously described by Fortin and Genest (*26*, *27*). Their studies showed a systematic positive bias of 3.0 µmol/L for the ADVIA Centaur, which according to the authors excludes the possibility of interchangeable use of the ADVIA Centaur and HPLC-FD methods. The reference ranges for Hcy concentration determined by both methods prove the correctness of this conclusion. These results show that the ADVIA Centaur method gave significantly higher Hcy values (mean: 12.6±3.3 with a central 95% range of 8.0–20.3 µmol/L) than the HPLC method (mean 9.5±2.5 with a central 95% range of 5.7–15.8 µmol/L) for the same patients samples (*26*). Referring the results obtained by us to those cited above, it can be stated that the systematic positive bias of 5.85 µmol/L for the CLIA method excludes the possibility of interchangeable use of the CLIA method with HPLC-FD.

The lack of specificity of the analytical method is most often the reason for the systematic bias. However, there are other possible reasons for the low agreement between the results obtained by the compared methods. Some of them are discussed below.

In the past poor quality reagents and access to inappropriate reference materials were a problem causing systematic errors. However, in this study equipment, reagents as well as controls and calibrators were provided by the kit manufacturers with the declared certificates (declaration of conformity according to directive 98/79 EC on in vitro diagnostic medical devices - CE IVD mark). Another one is that the Atellica IM HCY method, contrary to the HPLC-FD one, is fully automated. Hence, in the former the risk of error of the analyst performing the determination has therefore been significantly reduced. The possible limitation of the HPLC-FD method may be the potential risk with destruction of the fluorescent tHcy adduct by light after derivatization that could be the reason for the lower tHcy concentrations observed applying the HPLC-FD method. However, the results presented by Ubbink et al. showing great consistence of the results obtained by the HPLC-FD and GC-MS methods (the mean difference between the methods was - 1.42 µmol/L resulting in the mean proportional bias: - 6.2%) eliminated this possibility (*17*). In addition, to protect the samples against the light, the non-transparent dark vials were used provided by the kit manufacturer in which the samples were prepared for analysis to limit this potential source of error.

In the light of the above, it should be stated that the lower homocysteine values obtained by HPLC-FD compared to CLIA result from the greater selectivity of the first method, which makes it a preferential method for analysing the total concentration of homocysteine in the patient’s blood serum. This fact, however, imposes the need to set new reference ranges.
